# The association between Chinese eye exercises and myopia in children and adolescents: A systematic review and meta-analysis

**DOI:** 10.3389/fpubh.2023.950700

**Published:** 2023-03-10

**Authors:** Jie Tang, Yifei Pei, Jingjing Wang, Na Yan, Yunjiao Luo, Wen Zhou, Xiaojuan Wang, Wei Wang

**Affiliations:** ^1^School of Public Health, Xuzhou Medical University, Xuzhou, Jiangsu, China; ^2^Department of Ophthalmology, The Affiliated Xuzhou Municipal Hospital of Xuzhou Medical University, Xuzhou, Jiangsu, China; ^3^Key Laboratory of Human Genetics and Environmental Medicine, Xuzhou Medical University, Xuzhou, Jiangsu, China; ^4^Engineering Research Innovation Center of Biological Data Mining and Healthcare Transformation, Xuzhou Medical University, Xuzhou, Jiangsu, China

**Keywords:** myopia, Chinese, eye exercises, adolescents, meta-analysis

## Abstract

**Objective:**

This study aims to summarize the relevant evidence on the association between eye exercises and myopia in children and adolescents in China.

**Methods:**

The meta-analysis pooled the results of 12 studies, with a total of 134,201 participants. Another five studies (no OR for myopia as an outcome and meeting inclusion criteria) were reported in the systematic review. We searched PubMed, Web of Science, CNKI, Wan Fang, and reference lists of retrieved studies. Association estimates were pooled using random-effects meta-analyses. Odds ratios (ORs) and 95% confidence intervals (CIs) for eye exercises and myopia were pooled from a meta-analysis.

**Results:**

After standardizing the reference values, a pooled OR of the univariate analysis showed a 24% reduction in myopia in children and adolescents who performed eye exercises (OR = 0.76; 95% CI: 0.62–0.89). After adjusting the covariate, a pooled OR of multiple logistic analysis for myopia (OR = 0.87; 95% CI: 0.72–1.02) showed that there is no significance between eye exercises and myopia. However, in subgroup studies of the multivariate analysis, the large sample (OR = 0.84; 95% CI: 0.74–0.94) and Chinese database (OR = 0.80; 95% CI: 0.67–0.93) subgroup showed modest protective effects. In addition, five studies in the systematic review also evaluated the risk of myopia events, and Chinese eye exercises had a modest protective effect on myopic control, but the incorrect performance of and attitude toward eye exercises posed negative effects on their eyesight health.

**Conclusion:**

Chinese eye exercises have a modest protective effect on myopic control, but considering that the incorrect performance of and attitude toward eye exercises have a significant influence on the effect of eye exercises, the effect of eye exercises may not be enough to prevent the progress of myopia in the long term, and more standardized eye exercises need to be conducted.

## Introduction

Myopia is a refractive error in which light entering the eye parallel to the optical axis focuses in front of the retina when the human eye's regulation is relaxed (1). It is an important public health problem. There is increasing evidence showing that myopia not only causes blurred vision in teenagers but also reduces their learning efficiency and affects their school admission (2). It is also associated with an increased risk of several eye diseases, including myopic retinopathy, cataracts, glaucoma, visual impairment, and blindness (3–5). The prevalence of myopia has risen rapidly in recent decades, reaching epidemic levels among middle and high school students, renewing concerns about protecting the eyesight of Chinese students (6, 7).

The high incidence of myopia in China can be traced back to the early 1960s (8). To address this problem, according to the Chinese National Education Commission, Chinese students (around 6–17 years old) are required to perform eye exercises in school. Chinese eye exercises are developed based on traditional Chinese massage therapy and are performed in the form of self-massage of acupoints around the eyes. These exercises protect vision by improving blood circulation around the eyes, relaxing extra-ocular muscles, and reducing eye strain (9). The Chinese government has supported these eye exercises since 1963, believing they can prevent myopia in children. Over the past half-century, eye exercises have become a living habit among Chinese primary and secondary school students. At the same time, the prevalence of myopia among Chinese children has risen significantly in recent decades, reaching an epidemic level (30.1–78.4%) (10, 11). Therefore, eye exercises do not appear to play a critical role in preventing myopia or relieving eye fatigue (12).

Although some people believe that Chinese eye exercises may not be associated with the prevalence of myopia in children, others believe that the prevalence of myopia may be higher if children do not perform eye exercises. Some studies show that 90% of Chinese children do eye exercises every day, but they do not perform them correctly (13, 14). Most Chinese children cannot find accurate acupoints around their eyes, and they do not have accurate exercise pressure and operation skills. Recent studies show that Chinese eye exercises have a certain effect on relieving myopia symptoms in Chinese children (15–17). Some cross-sectional studies have found that there is a different correlation between the prevalence of myopia and eye exercises (18, 19). It has also been reported that performing eye exercises can seriously improve eyesight (20) and performing eye exercises incorrectly poses a non-trivial threat to vision health (19).

It remains to be confirmed whether the operation quality of Chinese eye exercises is poor, or whether the exercise itself affects the prevention of the progression of myopia. However, in the past decade, the number of observational studies has greatly increased, and the hypothesis that eye exercises can prevent myopia has been investigated. To the best of our knowledge, few systematic reviews or meta-analyses of the relationship between eye exercises and myopia have been published or registered to date. Using cross-sectional data, it is possible to determine the association between eye exercises and myopia, but causal relations between eye exercises and myopia could not be determined. In addition, several longitudinal cohort studies and clinical trials have recently been conducted to determine whether eye exercises have a protective effect on myopia. Therefore, we conducted a systematic review and meta-analysis to summarize and quantify all available evidence on the relationship between eye exercises and myopia.

## Methods and materials

### Search strategy

We searched several databases (PubMed, Web of Science, CNKI, and Wan Fang) from 2006 to 2021 to identify relevant articles (Supplementary Table S1). PubMed's keywords are retrieved based on a combination of the following three sections: (1) Myopia (myopia or myopic or shortsightedness or nearsightedness or refractive error); (2) Adolescent (youth or youths or adolescent or adolescents or teenager or teenagers or child or children or student or students); (3) Eye exercises (eye exercise or eye exercises). After the repeated articles were deleted, the retrieved works of literature were screened by title and abstract, and the full text was then extracted for evaluation. Finally, manual retrieval was carried out on the included references and related studies to supplement the missing literature retrieved from the electronic literature database. The search was performed by two investigators independently, and in instances of disagreement, a third investigator was consulted.

### Inclusion and exclusion criteria demographic characteristics

In the full article, these studies need to meet the following inclusion and exclusion criteria for meta-analysis purposes. The inclusion criteria were (1) reported myopia (prevalent or incident) as the outcome measure, (2) reported a measure of the association either as an effect estimate with 95% confidence interval (CI) or allowed for the calculation of it from raw data contained in the article, and (3) were limited to children and adolescents. We excluded animal studies and some studies that did not have a precise definition of myopia. We did not limit the studies based on the study design and may therefore have included interventional and observational studies. Studies that met our study questions but did not meet the criteria for meta-analysis were reported in our systematic review, listing reasons for exclusion, and documenting study limitations.

### Data extraction and quality evaluation

Two researchers extracted data and scored the quality of the included studies, respectively, and discussed the inconsistent results until an agreement was reached. The data types extracted include the name of the first author, year of publication, basic information about the study population (numbers and crowds), the region, the prevalence of myopia, the definition of myopia, odds ratios (ORs), and 95% CIs for myopia. The study quality was independently assessed by two evaluators using adapted Downs and Black lists (21). The original tool consisted of 27 items rated No/undetermined = 0 and YES = 1. The three additional items (9, 17, 22) on follow-up were not used to evaluate cross-sectional studies. Since a different number of items were used to evaluate each study, a percentage value (number of identified items/total number of items × 100) was assigned to each study. Study scores of ≥66.8% were considered high quality, 33.4–66.7% were considered moderate, and ≤ 33.3% were considered poor. Any differences between the two reviewers in data extraction and grading are resolved through discussion, using the original article as a reference.

### Statistical analysis

Stata 16.0 software was used for the meta-analysis, and odds ratios (ORs) and 95% CIs for myopia were selected as the combined effect scale (23). The random-effect model was used in the meta-analysis because of the expected heterogeneity in the research population, the definition of results, and the adjustment degree of confounding factors. At the same time, subgroup analysis and sensitivity analysis were carried out to explore the sources of heterogeneity (24). A funnel plot was drawn, and publication bias was detected by Begg's and Egger's tests (25).

## Results

### Literature screening process and results

**Figure 1:**
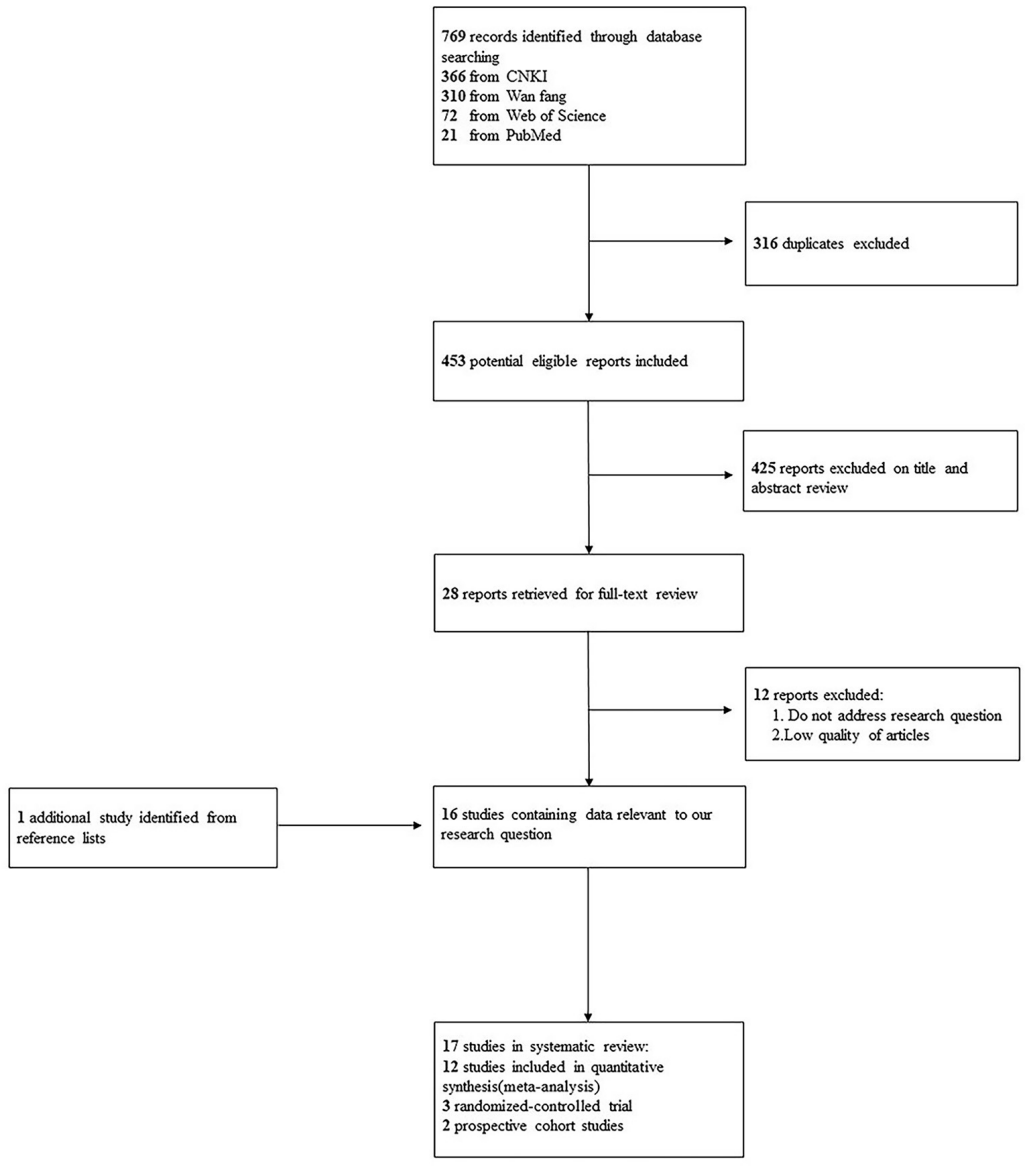
Flow diagram outlining the selection process for the inclusion of studies in the systematic review and meta-analysis.

We identified 769 articles from the database (366 from CNKI, 310 from Wan Fang, 72 from Web of Science, and 21 from PubMed). After 316 duplicates were excluded, there were 453 studies left (Figure 1). A further 425 irrelevant studies were removed by reading titles, abstracts, and keywords. The remaining 28 articles were reviewed for full-text reading, and 12 articles with low quality and other topics were excluded. Through manual retrieval of the included works of literature and related research by senior experts in this field, one related literature was added. The remaining 17 articles were searched by OR and 95% CI or computable related data, and 12 works of literature were finally included in the meta-analysis (15, 22, 26–36). For the remaining five studies (16–20), we were unable to perform a meta-analysis of studies with an endpoint of myopia progression because most studies failed to provide multivariate association measures that would allow us to pool estimates together.

### Characteristics of studies included in the meta-analysis

Characteristics of the 12 studies (15, 22, 26–36) included in the meta-analysis, such as the name of the first author, year of publication, basic information of study population (numbers and crowds), region, the prevalence of myopia, definition of myopia, odds ratios (ORs), and 95% CIs for myopia are summarized in Table 1. A total of 80,364 people were involved in the univariate analysis and 109,744 people were involved in the multivariate study. There were six studies with more than 2,000 participants, accounting for 50% of the total included literature. Seven studies were from Chinese databases, and five studies were from English databases. The studies estimated the prevalence of myopia in a wide range from 40.2 to 86.8%. All studies gave the response rate and described the sampling method, although the degree was different.

**Table 1:** Characteristics of the 12 studies included in the meta-analysis of eye exercises and myopia.

**References**	**Participant information**	**Study design**	**Univariate**			**Multivariate**						
			**OR (95% CI)**	***P***	**Adjust effect OR (95% CI)**	**OR (95% CI)**	***P***	**Adjust effect OR (95% CI)**	**Region**	**Quality**	**Myopia rate (%)**	**Definition of myopia**
Liu (31)	*n* = 1,700, middle school students	1. Cross-sectional study 2. Self-design questionnaire 3. Do not do eye exercises as a reference	0.605 (0.475–0.770)	< 0.001	N/A	No mention	No mention	N/A	Hubei	High	51.3	Visual acuity < 5.0
Wang and Lin (32)	*n* = 2,462, junior school students	1. Cross-sectional censuses 2. Self-design questionnaire 3. Do eye exercises as a reference	No mention	< 0.01	0.549 (0.303–0.994)	1.211 (1.015–1.446)	0.034	0.825 (0.69–0.90)	Xinjiang	High	55.5	Visual acuity < 5.0
Huang et al. (26)	*n* = 1,153, freshman students	1. Cross-sectional censuses 2. Self-design questionnaire 3. Do not do eye exercises as a reference	0.66 (0.45–0.97)	0.035	N/A	0.79 (0.51–1.21)	0.278	N/A	Nanjing	High	86.8	Self-report
Lin et al. (15)	*n* = 409, Chinese urban children	1. Cross-sectional study 2. Self-design questionnaire, CISS, and a cycloplegic autorefraction 3. Do not do eye exercises as a reference	0.62 (0.17–2.25)	0.55	N/A	1.46 (1.32–1.63)	0.1	N/A	Beijing	Medium	No mention	SE ≤ −0.5D
Lin et al. (22)	*n* = 836, Chinese rural children	1. Cross-sectional study 2. Self-design questionnaire, CISS, and a cycloplegic refraction 3. Do not do eye exercises as a reference	2.70 (1.80–4.04)	< 0.001	N/A	1.97 (1.19–3.26)	0.005	N/A	Beijing	Medium	No mention	SE ≤ −0.5D
Bian (35)	*n* = 31,080, primary school students	1. Cross-sectional censuses 2. Self-design questionnaire 3. Do not do eye exercises as a reference	No mention	No mention	N/A	0.709 (0.509–0.987)	0.042	N/A	Inner Mongolia	High	47.8	Visual acuity < 5.0
Shen and Li (36)	*n* = 889, high school students	1. Cross-sectional censuses 2. International standard questionnaire 3. Do not do eye exercises as a reference	No mention	0.01	0.481 (0.274–0.847)	0.41 (0.23–0.75)	< 0.01	N/A	Guangdong	Medium	53.7	Visual acuity < 5.0
Wang et al. (29)	*n* = 11,138, Inner Mongolia Medical Students	1. Cross-sectional censuses 2. Self-design questionnaire 3. Do eye exercises as a reference	1.18 (0.949–1.318)	< 0.001	N/A	1.29 (1.05–1.57)	0.01	0.775 (0.64–0.95)	Inner Mongolia	High	2011: 70.5, 2013: 69.2	Self-report
Kang et al. (27)	*n* = 260, school-age children	1. Nested case-control study 2. Self-design questionnaire 3. Do not do eye exercises as a reference	0.79 (0.41–1.53)	No mention	N/A	0.64 (0.27–1.47)	No mention	N/A	Beijing	High	54.2	SER ≤ −0.5D
Liu (30)	*n* = 11,400, elementary school students, middle school students, and high school students	1. Cross-sectional censuses 2. Self-design questionnaire 3. Do not do eye exercises as a reference	No mention	< 0.001	0.589 (0.543–0.638)	0.749 (0.67–0.84)	< 0.001	N/A	Wuhan	High	85.4	SER ≤ −0.5D
Yu (33)	*n* = 39,390, primary school students	1. Cross-sectional censuses 2. Self-design questionnaire 3. Do not do eye exercises as a reference	No mention	< 0.01	0.846 (0.773–0.925)	0.87 (0.78–0.97)	< 0.05	N/A	Shenzhen	Medium	40.2	Visual acuity < 5.0
Pan (34)	*n* = 10,727, middle school students and high school students	1. Cross-sectional study 2. Self-design questionnaire 3. Do not do eye exercises as a reference	No mention	< 0.05	0.792 (0.698–0.899)	1.034 (0.91–1.09)	>0.05	N/A	Ningbo	Medium	77.8	Visual acuity < 5.0

CI, confidence interval; SER, spherical equivalent refraction; OR, odds ratio; N/A, not adjusted; CISS, convergence insufficiency symptom survey.

### Pooled estimates of the association between Chinese eye exercises and prevalent myopia

**Figure 2:**
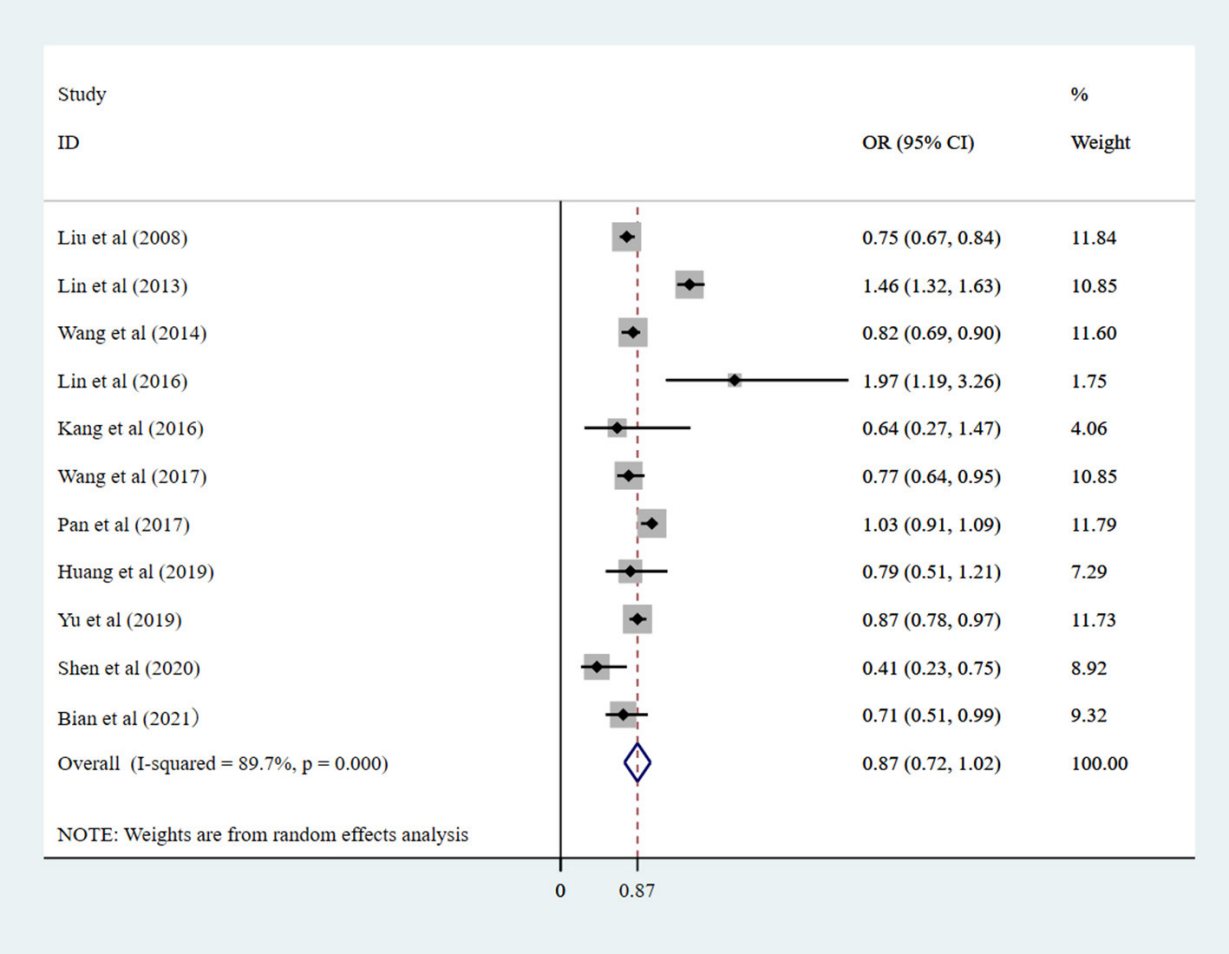
Randomized effect meta-analysis investigating the correlation between eye exercises and myopia in children and adolescents in multivariate analysis. CI, confidence interval; OR, odds ratio.

The heterogeneity test was carried out for these 12 studies, and there was obvious heterogeneity between the univariate and multivariate analyses. A univariate (*I*^2^ = 87.7%; *P*_heterogeneity_ = 0.000), multifactorial (*I*^2^ = 89.7%; *P*_heterogeneity_ = 0.000) random-effect model was used to combine the results. Pooled estimates are shown in Figure 2 and Supplementary Figure S1. In the univariate analysis, the random-effect meta-analysis showed that the pooled OR of myopia was 0.76 (95% CI: 0.62–0.89), and in the multivariate analysis, the random-effect meta-analysis showed that the pooled OR of myopia was 0.87 (95% CI: 0.72–1.02).

### Subgroup analyses

To investigate the source of heterogeneity, we used the published language, sample size (large sample: >2,000, small sample: ≤ 2,000), the definition of myopia, and quality as grouping variables in a prespecified subgroup analysis. The univariate analysis reveals that the heterogeneity of the language database subgroup and the quality subgroup has decreased slightly, indicating that the sources of heterogeneity may be the language database and the quality.

#### Language database group

**Figure 3:**
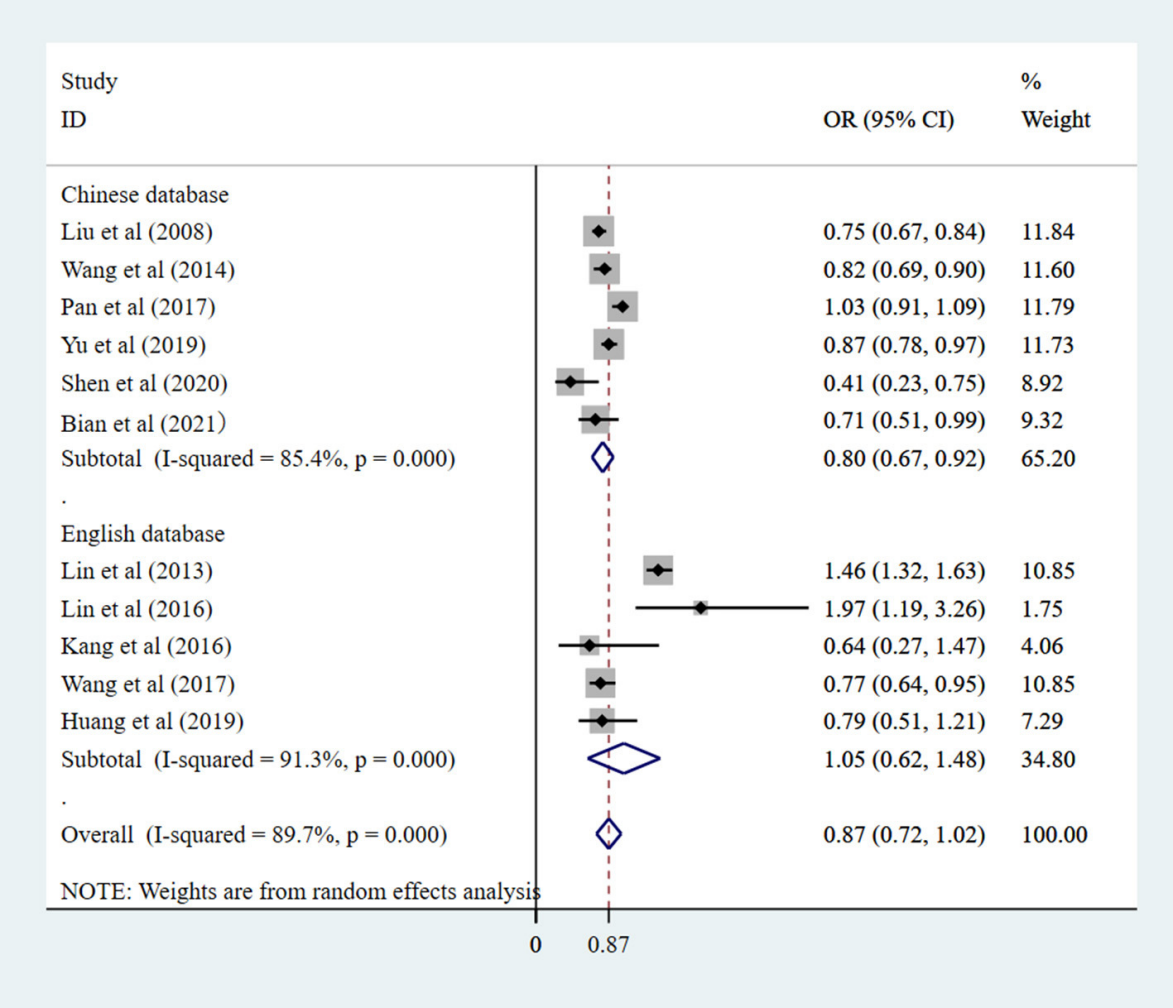
Language database subgroup of the studies on the correlation between eye exercises and myopia in children and adolescents in multivariate analysis.

As shown in Supplementary Figure S2 and Figure 3, in the univariate analysis, there are five studies from English databases, and the pooled OR was 1.04 (95% CI: 0.61–1.48). There were six studies from Chinese databases, and the pooled OR was 0.67 (95% CI: 0.54–0.80). There is greater heterogeneity among the Chinese databases studies [*I*^2^ = 87.6% (*P*_heterogeneity_ = 0.000) vs. *I*^2^ = 80.1% (*P*_heterogeneity_ = 0.000)]. In the multivariate analysis, there were five studies from English databases, and the pooled OR was 1.05 (95% CI: 0.62–1.48), and six studies from Chinese databases, and the pooled OR was 0.80 (95% CI: 0.67–0.93). There is greater heterogeneity among the English databases studies [*I*^2^ = 91.2% (*P*_heterogeneity_ = 0.000) vs. *I*^2^ = 85.5% (*P*_heterogeneity_ = 0.000)]. Chinese databases show that Chinese eye exercises had a protective modest effect on myopic control.

#### Sample group

**Figure 4:**
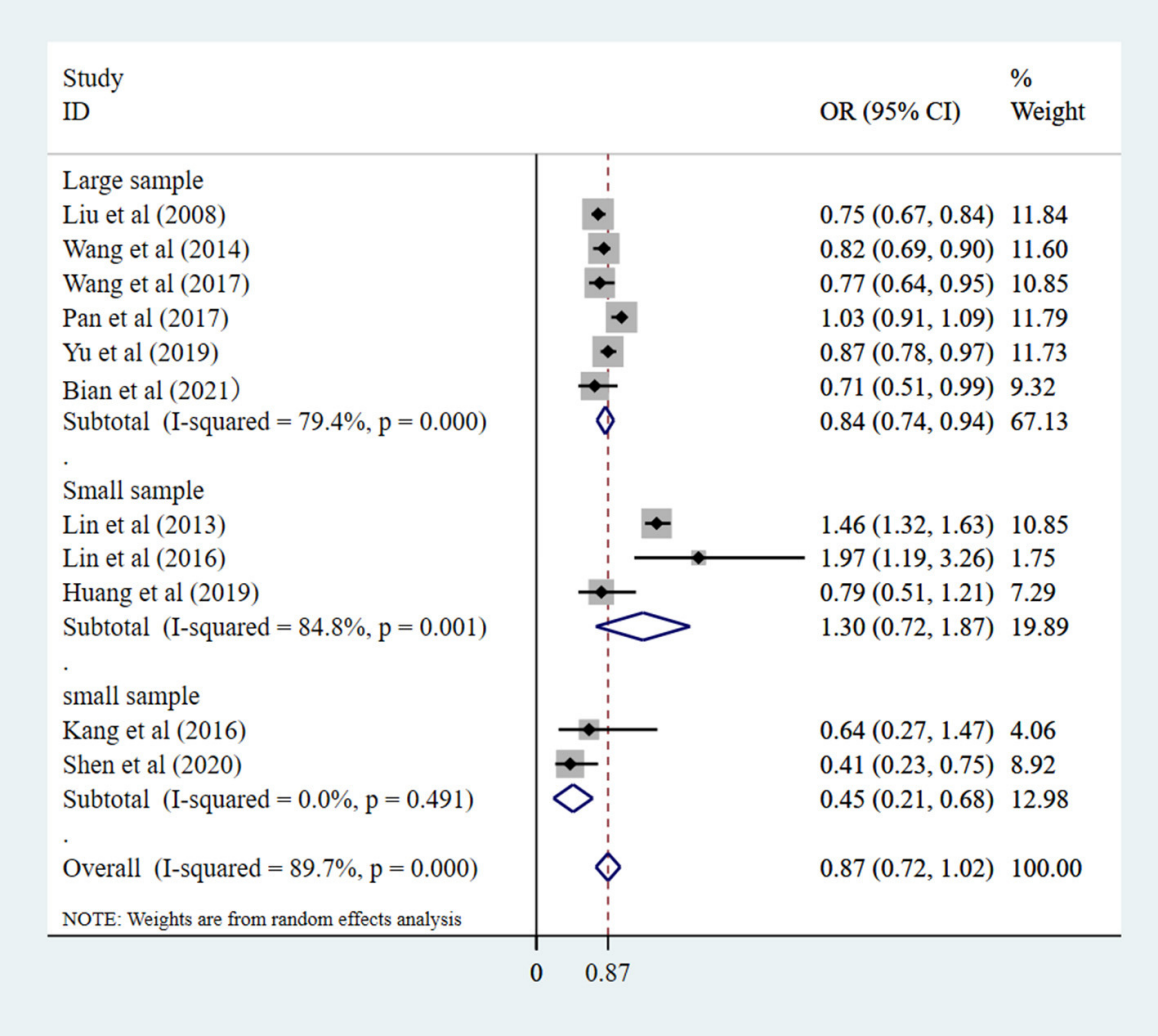
Sample size subgroup of the studies correlation between eye exercises and myopia in children and adolescents in multivariate analysis.

As shown in Figure 4 and Supplementary Figure S3, in the univariate analysis, there were five large sample studies with a pooled OR of 0.80 (95% CI: 0.62–0.98). There were six studies of the small sample, with a pooled OR of 0.71 (95% CI: 0.45–0.97). There was greater heterogeneity among large sample studies [*I*^2^ = 93.9% (*P*_heterogeneity_ = 0.000) vs. *I*^2^ = 65.9% (*P*_heterogeneity_ = 0.012)]. In the multivariate analysis, there were six large sample studies with a pooled OR of 0.84 (95% CI: 0.74–0.94) and five small sample studies with a pooled OR of 0.99 (95% CI: 0.42–1.55). There was also a greater heterogeneity among small sample studies [*I*^2^ = 92.8% (*P*_heterogeneity_ = 0.000) vs. *I*^2^ = 79.5% (*P*_heterogeneity_ = 0.000). Large sample studies show that Chinese eye exercises have a protective effect on myopic control.

#### Definition of myopia

As shown in Supplementary Figures S4, S5, in the univariate analysis, four studies define myopia as SER ≤ −0.5D [*I*^2^ = 78.7% (*P*_heterogeneity_ = 0.003)], and the pooled OR was 1.01 (95% CI: 0.37–1.66). In total, five studies define myopia as visual acuity < 5.0 [*I*^2^ = 71.9% (*P*_heterogeneity_ = 0.007)], and the pooled OR was 0.71 (95% CI: 0.58–0.83). In total, two studies define myopia as a self-report [*I*^2^ = 90.2% (*P*_heterogeneity_ = 0.001)], and the pooled OR was 0.93 (95% CI: 0.42–1.44). In the multivariate analysis, four studies define myopia as SER ≤ −0.5D [*I*^2^ = 95.5% (*P*_heterogeneity_ = 0.000)], and the pooled OR was 1.12 (95% CI: 0.58–1.66). In total, five studies define myopia as visual acuity < 5.0 [*I*^2^ = 85.2% (*P*_heterogeneity_ = 0.000)], and the pooled OR was 0.80 (95% CI: 0.65–0.96). In total, two studies define myopia as a self-report [*I*^2^ = 0.00% (*P*_heterogeneity_ = 0.918)], and the pooled OR was 0.77 (95% CI: 0.63–0.92).

#### Quality

As shown in Supplementary Figures S6, S7, in the univariate analysis, six studies were of high quality [*I*^2^ = 86.7% (*P*_heterogeneity_ = 0.000)], and the pooled OR was 0.73 (95% CI: 0.52–0.93). The five studies were of medium quality [*I*^2^ = 76.6% (*P*_heterogeneity_ = 0.002)], and the pooled OR was 0.79 (95% CI: 0.60–0.98). In the multivariate analysis, six studies were of high quality [*I*^2^ = 0.00% (*P*_heterogeneity_ = 0.908)], and the pooled OR was 0.77 (95% CI: 0.71–0.83). The five studies were of medium quality [*I*^2^ = 93.8% (*P*_heterogeneity_ = 0.000)], and the pooled OR was 1.02 (95% CI: 0.73–1.30).

### Sensitivity analyses

**Figure 5:**
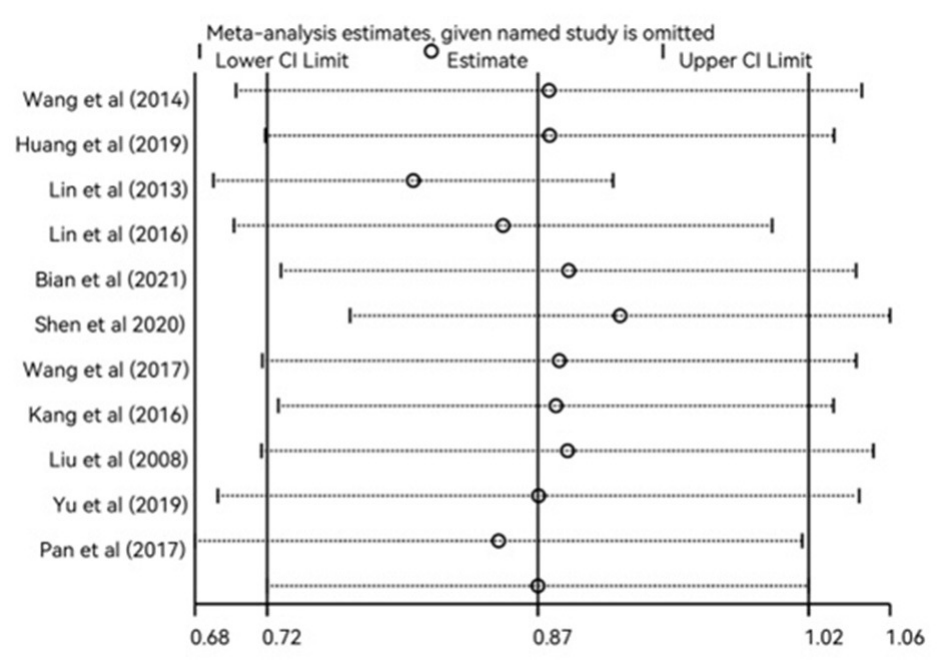
Sensitivity analysis of the relationship between eye exercises and myopia in children and adolescents in multivariate analysis.

To explore the source of heterogeneity, sensitivity analysis was conducted for the studies, and the included single studies were successively removed item by item. The results of the univariate analysis are shown in Supplementary Figure S8, and the results of the multivariate analysis are shown in Figure 5. Consistent with the original analysis results, single studies have little influence on the combined results, indicating that the combined effect value of this study is relatively stable.

### Assessment publication bias

**Figure 6:**
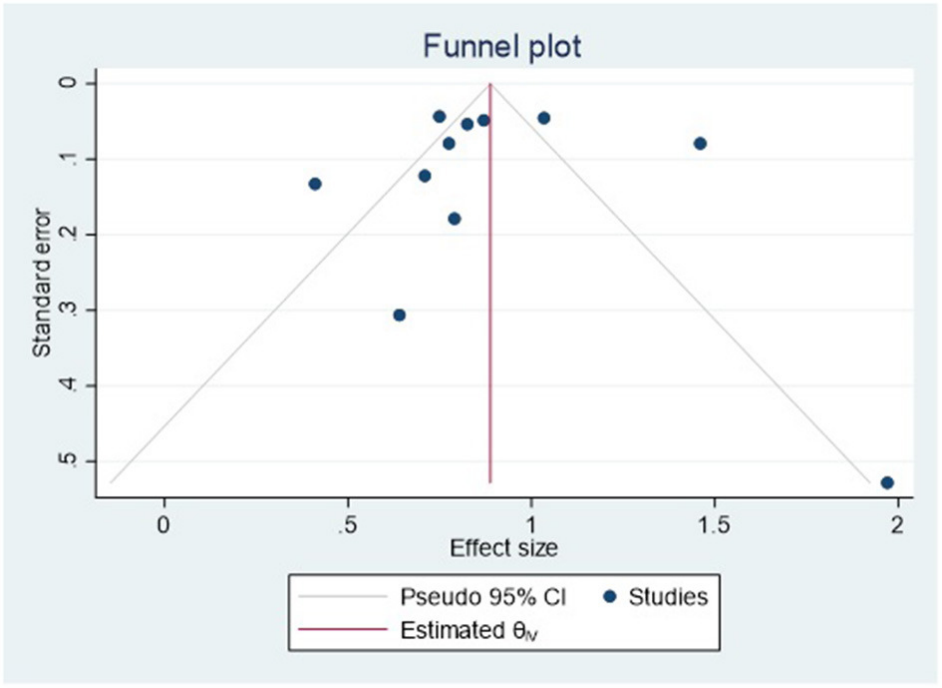
Publication bias funnel diagram of studies on the relationship between eye exercises and myopia in children and adolescents in multivariate analysis.

As shown in Figure 6 and Supplementary Figure S9, we assessed for publication bias in the 12 studies with a funnel plot; there are some signs of asymmetry in the figure, and the asymmetry in the distribution of studies contained in funnel plots may be due to publication bias, heterogeneity of effect sizes between studies or by chance. In univariate analysis, neither Begg's (z = 0.47, *P* = 0.64) nor Egger's (*t* = 1.52, *P* = 0.16) tests indicated publication bias in 11 studies. In the multivariate analysis, neither Begg's (z = 0.00, *P* = 1.00) nor Egger's (*t* = 0.52, *P* = 0.61) tests show any obvious publication bias (the results of Begg's and Egger's tests are primarily dependent on the *P*-value, and *P* < 0.05 is generally considered to be publication bias).

### Correlation estimates from other studies

Data from five studies involving 22,457 children were not available for meta-analysis (Table 2). The five studies investigated the relationship between eye exercises and myopia control (two prospective cohorts; three RCTs). Zhang et al. performed an RCT in China in which 405 students were in the same learning environment (20). They were divided into two groups according to whether they seriously performed eye exercises and analysis of the two groups of vision changes. The results showed that the attitude toward eye exercises had a significant impact on vision; unconscientiousness was associated with vision loss, *P* < 0.05, and RR was 2.41. Li et al. performed an RCT that enrolled 195 seventh graders and excluded five participants (three with a history of eye trauma and two with other interventions). They found that Chinese eye exercises temporarily relieved children's accommodative lag statistically, but it may not be clinically significant (16). The use of an instrumental variable method and bivariate probability method revealed the adverse effects of regular Chinese eye exercises on students' vision. Students who exercised regularly were 6.2 percentage points more likely to have vision impairment and 7.6% points more likely to be nearsighted than students who did not exercise regularly (19). The two cohort studies were followed at 9 months, 21 months, and 3 years. Wang et al. found no evidence that eye exercises had any effect on vision (18). Liang et al. found that Chinese eye exercises were effective in reducing myopia progression in urban Chinese students aged 6–17 years (17).

**Table 2:** Study on the relationship between eye exercises and myopia.

**References**	**Summary of study**	**Reason for exclusion from meta-analysis**	**Results**	**Biases and study limitations**
Wang et al. (18)	*n* = 11,934. A propensity-score-matched cohort study in 252 randomly-selected rural schools with baseline in September 2012 and follow-up surveys 9 and 21 months later.	No multivariate OR or RR was presented with myopia as an outcome	No evidence of an effect of eye exercises on change in vision or eyeglasses wear.	The follow-up time was long and the recall bias was large
Huang et al. (19)	School-based survey of 9,842 fourth-grade students was conducted using the instrumental variable (IV) method and a bivariate probability model to estimate the impact on students' visual acuity, as well as the incidence of visual impairment and myopia	Meets inclusion criteria	The commonly-observed incorrect performance of these eye exercises among Chinese students imposes non-trivial threats to their vision health.	1. Study is not a randomized controlled trial, which leaves room for estimation bias. 2. More specific and detailed measurement methods were not constructed. 3. The samples came from a single region.
Zhang and Tang (20)	405 students with the same learning environment were divided into two groups, according to whether they practiced eye exercises seriously and the visual acuity changes of the two health examinations were analyzed.	Meets inclusion criteria	Practice attitude has a significant influence on the effect of eye exercises. Unconscientiousness was associated with vision loss, P < 0.05. RR was 2.41	Small sample sizes
Li et al. (16)	*n* = 190. Randomized Controlled Trial, recruiting 195 grade 7 students and excluding 5 (3 for a history of ocular injury and 2 for receiving other excluding interventions)	No multivariate OR or RR was presented with myopia as an outcome. Meet inclusion criteria.	Chinese eye exercises, as performed daily in primary and middle schools in China, have statistically proven to be effective in preventing the progression of myopia, but in the long run, eye exercises may not be effective enough in preventing the progression of myopia.	Small sample sizes
Liang et al. (17)	*n* = 386. A 3-year cohort report from the Beijing Myopia Progression Study	Meets inclusion criteria	The Chinese acupoint eye exercises had a modest effect on reducing myopic progression among Chinese urban students aged 6–17 years.	Small sample sizes

CI, confidence interval; SER, spherical equivalent refraction; OR, odds ratio.

## Discussion

This systematic review and meta-analysis aimed to summarize all available pieces of evidence on the relationship between eye exercises and myopia control. A more comprehensive quantitative analysis of the association between eye exercises and myopia in children and adolescents was performed by meta-analysis, and the results of independent studies were statistically combined, taking into account the consistency of the data. Among the 17 included studies, a sample of 134,201 people was used for analysis, and 12 univariate and multivariate analyses were conducted. The results showed that if age, gender, and other factors were not considered, there would be a negative correlation between performing eye exercises and myopia. In other words, the probability of myopia decreased by 24% when eye exercises were done. If other variables were considered, there was no significance between doing eye exercises and controlling myopia. However, subgroup analyses of large samples and Chinese databases showed a moderate protective effect of Chinese eye exercises on myopia in multivariate analyses. This evidence suggests that eye exercises are effective in controlling myopia in large population studies.

Publication bias is a well-known flaw in meta-analysis methodology. The results of the subgroup analysis are not significant. The funnel chart shows some signs of asymmetry, which could be due to publication bias, heterogeneity, or the contingency of inter-study effects. However, due to the high representativeness of the sample, most studies control for confounding factors such as age, gender, and parental myopia, resulting in higher research quality in meta-analysis. Simultaneously, the qualitative sensitivity analysis of the included research reveals that the overall effect value is relatively stable. Furthermore, both Begg's and Egger's tests showed no significant publication bias.

The differences in the results of the multivariate analysis may be caused by the insufficient sample size and the non-standard attitude toward eye exercises. Most Chinese students only need to perform eye exercises one or two times a day for 5 min at school. Generally speaking, the time for eye exercises for Chinese children is very short. Moreover, most children and adolescents are unable to perform standard Chinese eye exercises. Previous research has shown that ~90% of Chinese children do not perform eye exercises correctly. Although they perform eye exercises every day, most of them cannot find the accurate acupoints around the eye, and there are no accurate pressure methods and manipulation skills (18, 37). Studies have shown that, compared with people who do not perform eye exercises regularly, people who perform eye exercises regularly have a 6.2% increase in the probability of vision damage and a 7.6% increase in the probability of myopia. Assuming that Chinese eye exercises are effective in protecting children's vision when used correctly (or at least do not pose a significant threat to children's visual health), we found that adverse effects may be caused by students performing eye exercises incorrectly (19). In addition, existing studies have proved that careful practice of correct eye exercises can make students take the initiative to rest the tired muscles with regular acupuncture points around their eyes (18, 37). Rhythmic massage can improve blood circulation in the eyes, accelerate muscle relaxation, enable blood vessels, nerves, and muscles to exert better physiological functions, gradually restore vision, and prevent vision loss (38). Therefore, children and adolescents should be encouraged to perform high-quality Chinese eye exercises. In addition, the protective effect of eye exercises on myopia may be related to better eye health awareness in children and adolescents who performed eye exercises. Kang et al. (27) conducted a 2-year study on 201 seventh-grade students and showed that students who can complete eye exercises with high quality have 0.15 D less myopia than other students. This may be because students who can correctly and conscientiously complete eye exercises are often able to pay more attention to eye protection, thus making it easier to develop good eye habits in life. As the Chinese government has been popularizing scientific ophthalmology knowledge and improving the eye health of children and adolescents, children's awareness of eye health has also been improved. They performed eye exercises with a serious attitude, and the protective effect of eye exercises was highly significant. Therefore, it is necessary for relevant departments and schools to further improve and strengthen the completion quality of eye exercises and improve the professionalism and responsibility of teachers and parents. Adhering to the scientific principle to continuously evaluate and optimize eye exercises, at the same time, we must fully implement policy intervention, pay equal attention to eye exercises and health education, form a multi-party linkage of “government-school-family,” and establish a scientific and effective myopia prevention and control system.

More definitive answers need to be provided by sufficiently reliable controlled trials. Given the widespread use of eye exercises in schools across China and existing policy practices supported by the Chinese government, it is unclear whether such research would be supported outside of China. A previous study (*n* = 190) reported that there is a statistical but not clinical significance between correct eye exercises and reduced accommodative lag in children (16). Accommodative lag is defined as the difference between accommodative demand and accommodative response, which may be related to myopia (39).

There are some shortcomings in the existing research on the relationship between eye exercises and myopia. (1) The reliability and validity of self-designed questionnaires are inconsistent. (2) Most of the current research focuses on the generalization of the relationship between eye exercises and myopia in children and adolescents. However, there is no research on whether there are differences in the relationship between eye exercises and myopia among different genders (boys and girls) and grades (primary school, junior high school, and senior high school). In addition, there is less research on the difference between eye exercises and myopia at different times and periods, and it is worth exploring further. (3) In the measurement of indexes, some studies did not conduct ciliary muscle paralysis before measuring myopia, which may overestimate the number of children and adolescents with myopia. Moreover, the collection of data on eye exercise performance by questionnaire could cause recall bias.

There are also some limitations in this review. (1) Literature retrieval is limited to English and Chinese, which may lead to language or cultural bias. (2) The prevalence of myopia in children and adolescents is highly heterogeneous, and the findings need to be validated with more high-quality studies. (3) Data were obtained from published literature, and some studies lacked extractable data, which limited the number of studies included in the meta-analysis. (4) These studies span a wide range of years and may have some influence on the results. All these limitations need to be filled by further research and exploration.

## Conclusion

Meta-analysis proves that Chinese eye exercises are effective in controlling myopia, but more high-quality studies, especially intervention studies, are needed to verify the reliability of the results. According to the results of this review, the prevention and control of myopia among children and adolescents need the joint participation of the government, schools, families, and students. In terms of suggestions, the government should improve the rules and regulations of myopia prevention and control, carry out quality education, and create a good learning atmosphere for students. Chinese schools should try their best to correct students' performance of Chinese eye exercises; neglecting hand hygiene can lead to conjunctivitis, and guidance on standard procedures should also be promoted, together with the school rules regarding eye exercises, for example, we should provide more guidance and instructions on how to do these exercises correctly and closely monitor students' performance. Students should improve their awareness of the severity of myopia, allocate their time reasonably, be skilled in the operation of acupoints, and take eye exercises seriously.

## Data availability statement

The original contributions presented in the study are included in the article/Supplementary material, further inquiries can be directed to the corresponding authors.

## Author contributions

WW was involved in conceptualization and methodology. JT and YP participated in data curation and writing the original draft. JW, NY, YL, and WZ supervised and validated the study. WW and XW participated in writing, reviewing, and editing. All authors contributed to the article and approved the submitted version.
