# Supplementary material for: The association between Chinese eye exercises and myopia in children and adolescents: A systematic review and meta-analysis
Source: Front Public Health. 2023 Mar 10;11:950700. doi: 10.3389/fpubh.2023.950700 (PMC10036375; doi:10.3389/fpubh.2023.950700)
Supplement: Supplementary file 1 [file Data_Sheet_1.docx]

*Supplementary* *appendix*

**Appendix 1.** Quality evaluation (Checklist for measuring study quality)

**Appendix 2.** Figure S1. Random-effects meta-analysis to investigate the correlation between eye exercises and myopia in children and adolescents in a univariate analysis.

**Appendix 3.** Figure S2. Language database subgroup of the studies on the correlation between eye exercises and myopia in children and adolescents in univariate analysis.

**Appendix 4.** Figure S3. Sample size subgroup of the studies correlation between eye exercises and myopia in children and adolescents in univariate analysis.

**Appendix 5.** Figure S4. Definition of myopia subgroup of the studies correlation between eye exercises and myopia in children and adolescents in univariate analysis.

**Appendix 6.** Figure S5. Definition of myopia subgroup of the studies correlation between eye exercises and myopia in children and adolescents in multivariate analysis.

**Appendix 7.** Figure S6. The quality subgroup of the studies correlation between eye exercises and myopia in children and adolescents in univariate analysis.

**Appendix 8.** Figure S7. The quality subgroup of the studies correlation between eye exercises and myopia in children and adolescents in multivariate analysis

**Appendix 9.** Figure S8. Sensitivity analysis of the relationship between eye exercise and myopia in children and adolescents in univariate analysis.

**Appendix 10.** Figure S9. Publication bias funnel diagram of studies on the relationship between eye exercises and myopia in children and adolescents in univariate analysis.

**Appendix 11.** Table S1. Search strategy

**Quality Evaluation**

**Checklist for measuring study quality**

**Reporting:**

1. Is the hypothesis/aim/objective of the study clearly described?

2. Are the main outcomes to be measured clearly described in the Introduction or Methods section?

3. Are the characteristics of the patients included in the study clearly described?

4. Does the study provide estimates of the random variability in the data?

5. Are the interventions of interest clearly described?

6. Are the distributions of principal confounders in each group of subjects to be compared clearly described?

7. Are the main findings of the study clearly described?

8. Have all important adverse events that may be a consequence of the intervention been reported?

9. Have the characteristics of patients lost to follow-up been described？

10. Have actual probability values been reported (e. g. 0.035 rather than <0.05) for the main outcomes except where the probability value is less than 0.001?

**External validity**

11. Were the subjects asked to participate in the study representative of the entire population from which they were recruited?

12. Were those subjects who were prepared to participate representative of the entire population from which they were recruited?

13. Were the staff, places, and facilities where the patients were treated, representative of the treatment the majority of patients receive?

**Internal validity - bias**

14. Was an attempt made to blind study subjects to the intervention they have received?

15. Was an attempt made to blind those measuring the main outcomes of the intervention?

16. If any of the results of the study were based on "data dredging", was this made clear?

17. In trials and cohort studies, do the analyses adjust for different lengths of follow-up of patients, or in case-control studies, is the time period between the intervention and outcome the same for cases and controls?

18. Were the statistical tests used to assess the main outcomes appropriate?

19. Was compliance with the intervention/s reliable?

20. Were the main outcome measures used accurate (valid and reliable)?

**Internal validity - confounding (selection bias)**

21. Were the patients in different intervention groups (trials and cohort studies) or were the cases and controls (case-control studies) recruited from the same population?

22. Were study subjects in different intervention groups (trials and cohort studies) or were the cases and controls (case-control studies) recruited over the same period of time?

23. Were study subjects randomized to intervention groups?

24. Was the randomized intervention assignment concealed from both patients and health care staff until recruitment was complete and irrevocable?

25. Was there adequate adjustment for confounding in the analyses from which the main findings were drawn?

26. Were losses of patients to follow-up taken into account?

**Power**

27. Did the study have sufficient power to detect a clinically important effect where the probability value for a difference being due to chance is less than 5%?

**Figure S1. Random-effects meta-analysis to investigate the correlation between eye exercises and myopia in children and adolescents in a univariate analysis.**

***Note*: CI: confidence interval; OR: odds ratio**

**Figure S2.** **Language database subgroup of the studies on the correlation between eye exercises and myopia in children and adolescents in univariate analysis.**

***Note*: CI: confidence interval; OR: odds ratio**

**Figure S3.** **Sample size subgroup of the studies correlation between eye exercises and myopia in children and adolescents in univariate analysis.**

***Note*: CI: confidence interval; OR: odds ratio**

**Figure S4. Definition of myopia subgroup of the studies correlation between eye exercises and myopia in children and adolescents in univariate analysis.**

***Note*: CI: confidence interval; OR: odds ratio**

**Figure S5. Definition of myopia subgroup of the studies correlation between eye exercises and myopia in children and adolescents in multivariate analysis.**


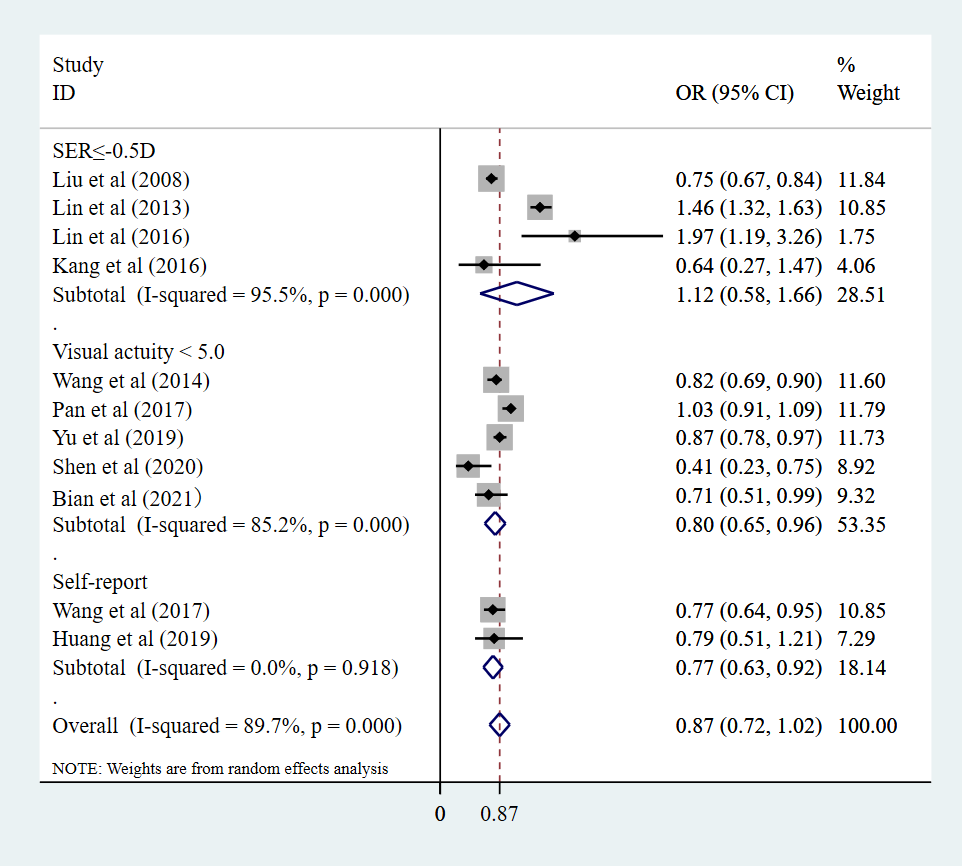


***Note*: CI: confidence interval; OR: odds ratio**

**Figure S6. The quality subgroup of the studies correlation between eye exercises and myopia in children and adolescents in univariate analysis.**

***Note*: CI: confidence interval; OR: odds ratio**

**Figure S7. The quality subgroup of the studies correlation between eye exercises and myopia in children and adolescents in** **multivariate analysis**


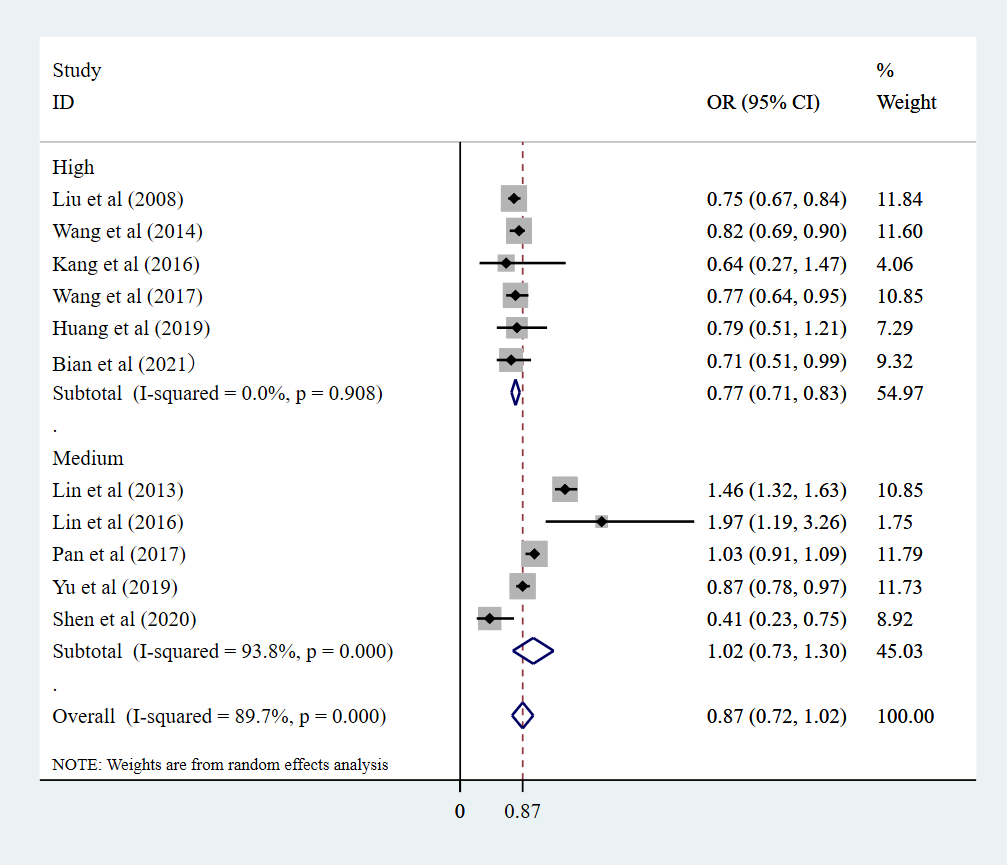


***Note*: CI: confidence interval; OR: odds ratio**

**Figure S8.** **Sensitivity analysis of the relationship between eye exercise and myopia in children and adolescents in** **univariate analysis.**


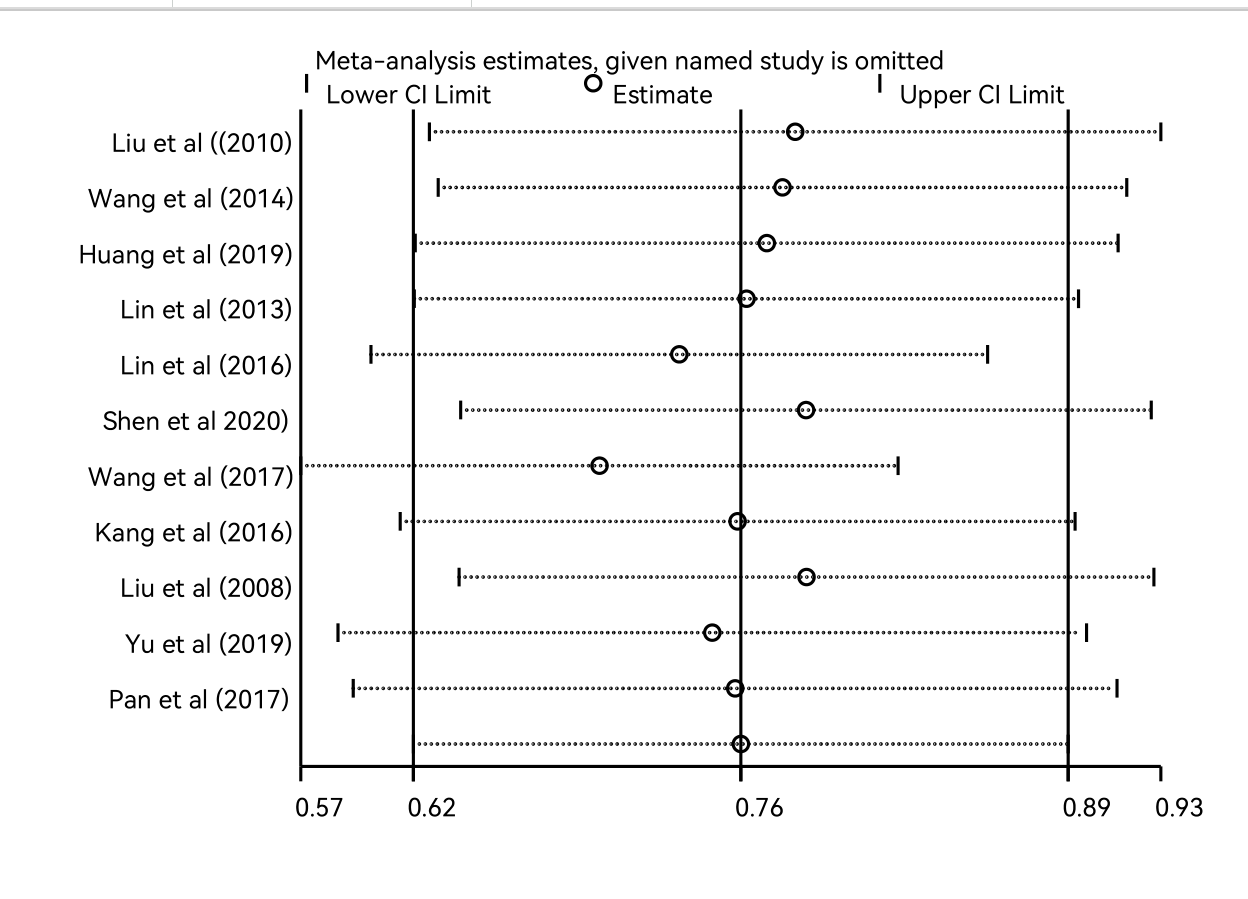


**Figure S9. Publication bias funnel diagram of studies on the relationship between eye exercises and myopia in children and adolescents in univariate analysis.**

**Table S1. Search strategy**

| **Search number** | **Search Details** |
| --- | --- |
| **PubMed** |  |
| 4 | (((((((Myopia [Title/Abstract]) OR (myopia)) OR (myopic)) OR (shortsightedness)) OR (nearsightedness)) OR (refractive error)) AND (((((((Adolescent[Title/Abstract]) OR (youth)) OR (youths)) OR (adolescent)) OR (adolescents)) OR (teenager)) OR (teenagers))) AND (((Eye exercises[Title/Abstract]) OR (eye exercise)) OR (eye exercises)) |
| 3 | ((Eye exercises [Title/Abstract]) OR (eye exercise)) OR (eye exercises) |
| 2 | ((((((Adolescent [Title/Abstract]) OR (youth)) OR (youths)) OR (adolescent)) OR (adolescents)) OR (teenager)) OR (teenagers) |
| 1 | (((((Myopia [Title/Abstract]) OR (myopia)) OR (myopic)) OR (shortsightedness)) OR (nearsightedness)) OR (refractive error) |
| **Web of Science** |  |
| 4 | ((#1) AND #2) AND #3 |
| 3 | (TS= (Eye exercises )) OR TS=(eye exercises) |
| 2 | ((((((((((TS=(Adolescent)) OR TS=(youth)) OR TS=(youths)) OR TS=(adolescent)) OR TS=(adolescents)) OR TS=(teenager)) OR TS=(teenagers)) OR TS=(child)) OR TS=(children)) OR TS=(student)) OR TS=(students) |
| 1 | ((((TS=(Myopia)) OR TS=(myopic)) OR TS=(shortsightedness)) OR TS=(nearsightedness)) OR TS= (refractive error) |
| **CNKI** | Check Chinese and English extensions. |
| 2 | （眼保健操（主题）OR 屈光不正（主题））AND 近视（主题） |
| 1 | 眼保健操（主题）OR 屈光不正（主题） |
| **Wan Fang** | Check Chinese and English extensions. |
| 2 | （眼保健操（主题）OR 屈光不正（主题））AND 近视（主题） |
| 1 | 眼保健操（主题）OR 屈光不正（主题） |
